# Facing the Pandemic in Italy: Personality Profiles and Their Associations With Adaptive and Maladaptive Outcomes

**DOI:** 10.3389/fpsyg.2022.805740

**Published:** 2022-02-24

**Authors:** Flavia Cirimele, Concetta Pastorelli, Ainzara Favini, Chiara Remondi, Antonio Zuffiano, Emanuele Basili, Eriona Thartori, Maria Gerbino, Fulvio Gregori

**Affiliations:** Department of Psychology, Sapienza University of Rome, Rome, Italy

**Keywords:** COVID-19 quarantine, person-oriented approach, young adults, prosocial behavior, interpersonal aggression, depressive symptoms, anxiety problems

## Abstract

The negative impact of the COVID-19 pandemic on individuals’ psychosocial functioning was widely attested during the last year. However, the extent to which individual differences are associated with adaptive and maladaptive outcomes during quarantine in Italy remains largely unexplored. Using a person-oriented approach, the present study explored the association of personality profiles, based on three broad individual dispositions (i.e., positivity, irritability, and hostile rumination) and two self-efficacy beliefs in the emotional area (i.e., expressing positive emotions and regulating anger emotion), with adaptive and maladaptive outcomes during the first Italian lockdown (March–June 2020). In doing so, we focused also on how different age groups (i.e., young adults and adults) differently faced the pandemic. The study was conducted through an online survey from May to June 2020 and included 1341 participants living in Italy, divided into two groups: 737 young adults aged 18–35 and 604 adults aged 36–60 years old. Latent Profile Analysis identified three personality profiles: resilient, vulnerable, and moderate. A subsequent path analysis model showed that the resilient profile was positively associated with prosocial behavior as an indicator of adaptive outcome, and negatively associated with three maladaptive outcomes: interpersonal aggression, depressive symptoms, and anxiety problems. Contrarily, the vulnerable profile resulted negatively associated with prosocial behavior and positively associated with the three maladaptive outcomes. Finally, regarding age group differences, young adults belonging to the vulnerable profile showed a greater association especially with interpersonal aggression, depression, and anxiety problems, as compared to adults belonging to the same profile. Overall, the results of the present study highlighted the importance to analyze individual functioning during an isolation period by using a person-oriented approach. Findings evidenced the existence of three different profiles (i.e., Resilient, Vulnerable, and Moderate) and subsequent path analysis revealed, especially for the vulnerable profile and young adults, a greater maladaptive consequence of the quarantine. The practical implications will be discussed.

## Introduction

In February 2020, Italy became the first and most affected country in Europe by the COVID-19 pandemic. In response to the ongoing public health emergency, the Italian government implemented strong containment measures, such as self-isolation and social distancing and the complete closure of schools, commercial stores, and public offices. Also, due to the high percentage of COVID-related mortality and the lack of adequate knowledge about the COVID-19 virus, Italians experienced concerns and worries about both their own and their relatives’ physical and mental health. Although the COVID-19 preventive measures guaranteed protection in terms of contagious’ spread and sustainability of national health services (Anderson et al., 2020), long-term isolation negatively affected individuals’ physical and psychological wellbeing (e.g., Phiri et al., 2021). For instance, previous studies conducted during this first lockdown period in Italy documented an increase in symptoms of depression, anxiety, and sleep disorders as compared to the period before the quarantine (e.g., Cellini et al., 2020), especially for young adults (e.g., Di Giuseppe et al., 2020).

Thus, the containment measures implemented during the COVID-19 pandemic caused a wide range of reactions. According to some authors (e.g., Harriger et al., 2021), while some people reported maladaptive problems (i.e., anxiety, depression, aggression) in response to the uncertainty and extraordinary preventive measures implemented during COVID-19, other people put into action adaptive responses mostly associated to the welfare of others. In this vein, prosocial-oriented actions, such as the desire to help, comfort, and care about others in need might represent an alternative response to COVID-19 related stress.

In our study, we wanted to advance knowledge on the role of personality characteristics in facing the psychological effect of the COVID-19 pandemic in Italy. We used a person-oriented approach to identify groups of individuals who share a set of individual characteristics that may protect from or exacerbate the psychological impact of COVID-19. The person-oriented approach is the most suitable framework and technique to capture the uniqueness of individuals in terms of understanding the dynamic process of interaction of operating factors within individuals and seems to be more valuable in contributing to the explanation of individual behaviors (Bergman and Magnusson, 1997; Bergman et al., 2003). Accordingly, we selected a set of personality predispositions (i.e., positivity, irritability, and hostile rumination) and a set of self-efficacy in the emotional domain (i.e., expressing positive emotions and regulating anger emotion) that have been shown to function as risk and protective factors for individual adjustment (Caprara and Cervone, 2000; Caprara and Steca, 2007; Caprara, 2015).

Overall, we explored patterns of personality profiles and tested their associations with adaptive (i.e., prosocial behavior) and maladaptive (i.e., interpersonal aggression, depressive symptoms, and anxiety problems) outcomes among Italian young adults and adults.

### Personality Dispositions and Self-Efficacy Beliefs

According to the interactionist perspective (Bandura, 1986; Magnusson, 1998), human functioning and behaviors are interrelated with social experiences and environmental factors. Individual characteristics are differentiated and act interdependently in a complex system influenced by life experiences and behaviors. In order to capture different aspects of individual functioning, it is crucial to consider both individual dispositions and individual perceptions of agency and capabilities (Caprara and Cervone, 2000; Caprara et al., 2008; Mischel and Shoda, 2008).

Based on this theoretical premise, we aimed to explore different configurations of personality characteristics by focusing on (1) three individual dispositions, such as positivity, irritability, and hostile rumination, and (2) two facets of self-efficacy beliefs in the emotional domain, such as self-efficacy in expressing positive emotions and self-efficacy in regulating anger during quarantine.

First, in regards to individual dispositions, several studies conducted before the pandemic demonstrated the unique beneficial role of positivity on psychosocial functioning (e.g., Caprara et al., 2019). Positivity represents an enduring dispositional self-evaluative tendency to view oneself, life, and the future under a positive outlook (Caprara et al., 2012), and played a beneficial role in people’s wellbeing (see Caprara et al., 2019, for a review). For example, cross-sectional (e.g., Zuffianò et al., 2019) and longitudinal (e.g., Luengo Kanacri et al., 2017) studies demonstrated the positive association between positivity and prosociality in late childhood and adolescence. Positivity also resulted negatively associated with anxiety, depression, and aggressive behaviors from late childhood (Zuffianò et al., 2019) through the elderly period (e.g., Borsa et al., 2016; Caprara M. et al., 2017). Moreover, irritability, or the individual tendency to react impulsively and rudely at the slightest provocation (Caprara et al., 1985), and Hostile Rumination, or the individual tendency to store ill feelings, expectations, attributions, and desires for vengeance after self-threatening provocation (Caprara et al., 1986), have been related to exacerbation of aggressive behaviors (e.g., Caprara et al., 2007), to respond to threatening stimuli in a reactive and impulsive manner (e.g., Bettencourt and Kernahan, 1997), and to manifest higher levels of anxiety and emotional instability problems (e.g., Butler and Nolen-Hoeksema, 1994; Nolen-Hoeksema et al., 1994).

Second, in regards to the emotion regulation domain, we considered self-efficacy in expressing positive emotions, or the individual perception to be capable to express positive emotions, such as joy, happiness, and satisfaction (Caprara and Gerbino, 2001), and self-efficacy in regulating anger, which is the individuals’ belief to be capable to adequately regulate their anger under several challenging circumstances (Caprara and Gerbino, 2001). These two aspects of individuals’ beliefs in the emotional domain resulted strictly associated on the one side, with a higher tendency to engage in prosocial behaviors (e.g., Caprara and Steca, 2007), and on the other, to a lower tendency toward aggressive behaviors, anxiety and depression (e.g., Caprara et al., 2008).

Based on these premises, we aimed to explore how these pivotal personality characteristics (i.e., positivity, irritability, hostile rumination, self-efficacy beliefs in expressing positive emotions, and self-efficacy beliefs in managing anger) interact with each other during the COVID-19 pandemic. To our knowledge, no previous studies investigated patterns of personality profiles and their association to individual adjustment in a novelty and challenging situation as the first lockdown in Italy.

### Adaptive and Maladaptive Outcomes During COVID-19 Pandemic

An increasing number of studies have examined the negative effects of COVID-19 lockdown on psychosocial functioning (e.g., Gloster et al., 2020), such as adaptive (i.e., prosocial behavior) and maladaptive (i.e., interpersonal aggression, depressive symptoms, and anxiety problems) responses.

#### Prosocial Behavior

Prosocial behaviors were generally defined as voluntary actions aimed to benefit others (e.g., Eisenberg, 2006). The role that these kinds of positive behaviors play within societies is not a novelty in the literature. For example, Luengo Kanacri et al. (2014) showed that in the transition to adulthood, prosocial Italian youth are more likely to engage in civic actions. The beneficial influence of prosocial behavior on civic engagement was also highlighted during the COVID-19 pandemic. In fact, some individuals have shifted from a self to a more collectivistic view (e.g., Ling et al., 2020), in which long-term collective interests depend on the respect of social norms dictated by COVID-19 pandemic restrictions.

Considering that prosocial behavior is related to self-transcendent values (i.e., values that emphasize concern for the welfare and interest of others, such as, e.g., benevolence and universalism; Schwartz, 2010) and other-oriented dispositions (i.e., agreeableness; Caprara and Steca, 2007), scholars stressed that individuals’ compliance with government’s restrictions and recommendations (i.e., wearing masks, social distancing) could be considered as forms of prosocial behaviors. Likely, recent results from a study by Campos-Mercade et al. (2021) conducted with Swedish adults showed that prosociality predicted health behaviors during the COVID-19 pandemic. In detail, people with higher levels of prosocial behavior showed a high tendency to follow governments norms that reduced contagious’ spread (i.e., buying face masks, staying at home, and maintaining physical distancing). Moreover, significant associations were found between higher levels of individuals trust in government actions determined by clear messages regarding the pandemic trend or well-organized process aimed to reduce COVID-19 spread, and prosocial behavior during the pandemic, also across different societies (e.g., Han et al., 2021; Romano et al., 2021).

Since prosocial behavior seems to play an adaptive role during the COVID-19 emergency, identifying configurations of personality profiles associated with prosocial behavior during the COVID-19 pandemic may be informative regarding individuals’ predisposition to engage in collectivistic actions during a pandemic.

#### Interpersonal Aggression

Aggressive behaviors can be defined as those behaviors aimed at physically or verbally hurting others, namely, aggressive behaviors (Caprara and Pastorelli, 1989). Some recent studies have shown an increase in interpersonal aggression and aggressive behaviors during the COVID-19 pandemic. For example, a longitudinal study conducted on Italian young adults showed an increasing tendency to enact different forms of aggressive behaviors (e.g., blaming others or screaming a lot) across the first 4 weeks of the Italian lockdown (Parola et al., 2020). Moreover, Deng and Feng (2021) showed that in the Hubei, the most affected province of China by the COVID-19, a higher level of life satisfaction (e.g., conceptualized as individual strength) buffered the relationship between perceived threat of COVID-19 and aggressive tendencies during the health emergency. Studies have also shown that aggressive behaviors during COVID-19 occurred through online communications (e.g., Chu et al., 2021; Pascual-Ferrá et al., 2021; Ye et al., 2021), suggesting the importance of considering internet-based communication experiences when assessing for aggressive behaviors in the era of COVID-19.

#### Depressive Symptoms and Anxiety Problems

Depressive and anxiety problems can be defined as emotional problems concerning the manifestation of mood deflection, worries, sadness, and guilt that tend to frequently appear together (Weissman et al., 1999; Graber and Sontag, 2009). The COVID-19 pandemic has increased fear of infection and worries among the general population. Also, long-term isolation and a high alert period exacerbated feelings of loneliness and symptoms of anxiety and depression (e.g., Deng et al., 2020; Lakhan et al., 2020). Results of several reviews indicated an increase of these symptoms since the beginning of the health emergency. For instance, a review by Deng et al. (2020) attested a prevalence of anxiety and depression during the pandemic between 45 and 47% in Ecuador, China, Iran, Italy, and Turkey. Across studies conducted from December 2019 to June 2020 in Italy, Spain, Iran, India, and China, Lakhan et al. (2020) found that people experienced 35% of anxiety problems and 20% of depressive symptoms. Similar results were found in a review of Luo et al. (2020), which included 62 studies conducted from November 2019 to May 2020 with samples from China, Iran, Italy, Spain, and Turkey. In detail, across these meta-analytic studies, results showed a prevalence of 33% of depression and 28% of anxiety symptoms, which were exacerbated in the case of coronavirus infections, reaching 55% of depression and anxiety prevalence. Studies conducted with only the Italian population showed similar alarming rates since the beginning of the first Italian lockdown (i.e., March–June 2020; e.g., Rossi et al., 2020). For example, Mazza et al. (2020) through an online survey with 2766 participants, identified that 19% of individuals reported high levels of anxiety problems and 32% high depressive symptoms. Taken together, these findings suggest a steady increase of depressive symptoms and anxiety problems in times of pandemic.

### Young Adults and Adults Differences in Facing the COVID-19 Pandemic

From a developmental point of view (e.g., Arnett, 2006), it is possible to consider different developmental stages across adulthood, with specific characteristics, demands, and challenges. In this view, young adults are generally defined as individuals between 18− and 29-year-olds involved in a process to become adults characterized by identity changes and explorations. However, cultural and socioeconomic factors could influence this transition period (Mary, 2014). For example, Italians young adults showed lower levels of emerging adulthood dimensions (i.e., entry to the labor market, parenthood, marriage) compared to populations with similar socioeconomic characteristics (e.g., Crocetti et al., 2015).

Despite some studies evidenced heterogeneous reactions of young adults during the COVID-19 pandemic (e.g., Harriger et al., 2021; Truskauskaite-Kuneviciene et al., 2021), a growing amount of studies attested a greater negative impact of COVID-19 quarantine especially on young adults (e.g., Ohannessian, 2021). For example, several findings showed that young adults reported higher depressive symptoms and anxiety problems than adults and older adults in the period related to the health emergency (e.g., Ahmed et al., 2020; Gao et al., 2020; Huang and Zhao, 2020; Lei et al., 2020; Ozamiz-Etxebarria et al., 2020; Perveen et al., 2020). Negative correlations were found between age and levels of depression and anxiety during the COVID-19 pandemic (e.g., Panchal et al., 2020; Solomou and Constantinidou, 2020). Finally, regarding aggressive behavior, Parola et al. (2020), using an Italian sample, showed an increase in frequencies to behave aggressively toward others from their first to the fourth week of lockdown in young adults. Overall, these findings showed that, during the COVID-19 pandemic, young adults resulted more compromised in developing maladaptive problems compared to adults.

Accordingly, in the present study, we explored patterns of personality profiles and tested their associations with adaptive (i.e., prosocial behavior) and maladaptive (i.e., interpersonal aggression, depressive symptoms, and anxiety problems) outcomes among young adults and adults.

### The Present Study

Despite the growing number of studies investigating the effects of restrictive measures due to the COVID-19 pandemic, the extent to which different configurations of individual dispositions and self-efficacy beliefs were associated with adaptive and maladaptive outcomes during this period remains largely unexplored. Following previous conceptualizations of personality functioning (e.g., Caspi et al., 2005), we adopted a person-oriented approach, which is more informative in terms of patterns of individual functioning. The person-oriented approach allows us taking into account more precisely oscillations in single individual dispositions that operate concurrently with other personality characteristics that, in turn, affect individuals’ behaviors and adjustment (e.g., Magnusson, 1998; Caspi et al., 2005).

The aim of the present study is threefold.

First, to identify personality profiles based on three broad dispositional tendencies (i.e., positivity, irritability, and hostile rumination) and two self-efficacy beliefs in the emotional domain (i.e., self-efficacy in expressing positive emotions and self-efficacy in regulating anger) in Italy during the first lockdown (March–June 2020). Based on several previous studies investigating patterns of individual functioning based on personality characteristics (e.g., Isler et al., 2017), we expect to find at least two profiles: a well-adapted profile characterized by higher emotional regulation (i.e., higher scores in emotional self-efficacy), higher positivity, and lower reactive or negative responses to threatening situations (i.e., lower scores in irritability and hostile rumination); and a more compromised profile, characterized by a lower emotion regulation (i.e., lower scores in emotional self-efficacy), lower positivity, and a higher tendency to react toward environmental stimulus with anger and hostility (i.e., higher irritability and hostile rumination).

Second, to examine the associations among emerged personality profiles and the occurrence of prosocial behavior, interpersonal aggression, depressive symptoms, and anxiety problems during the first lockdown in Italy (March–June 2020). We expect to find that a well-adapted profile will be associated with a better adjustment during quarantine (e.g., higher frequency to behave prosocially, lower interpersonal aggression, and lower depression and anxiety), while a compromised profile will be more associated with maladjustment during quarantine (e.g., lower prosocial behaviors, higher interpersonal aggression, and higher depression and anxiety).

Third, to examine the moderating role of age (young adults vs. adults) both in the personality profile configurations and in their associations with adaptive and maladaptive outcomes. In detail, since previous studies evidenced the challenging and demanding period of young adults (e.g., Arnett et al., 2014) and its higher impairment compared to adults in coping with the challenges associated with the COVID-19 pandemic (e.g., Parola et al., 2020), we hypothesiz that young adults will be more compromised than adults in both personality profiles and in facing the challenging of COVID-19 pandemic.

Finally, since previous studies showed that women were more compromised compared to men in maladaptive outcomes (e.g., Lei et al., 2020; Mazza et al., 2020) and that individual who was certainly or uncertainly exposed to COVID-19 infection scored lower in psychological wellbeing (e.g., Favieri et al., 2021), we control for gender and exposure to COVID-19 covariates in our analysis.

## Materials and Methods

### Participants

Participants were drawn for a wider project entitled “Facing with COVID-19: The role of individual resources and new technologies,” aimed to investigate the effects of COVID-19 pandemic on individual’s psychological wellbeing, as well as the impact of new technologies—use and increase in that use—in the Italian population. We considered 1341 participants (33% men) from 18 to 60 years old (*M*age = 36.88; SD = 12.22). To respond to the third aim of the present study, we divided our total sample into two different age groups, the first group (55% of the total sample) in which we categorized subjects from 18 to 35 years old (*M*age = 27; 30% males), and the second group (the 45% of the total sample) in which we categorized subjects from 36 to 60 years old (*M*age = 49; 36% males). We refer to young adults for the 18–35-year-olds groups and adults for the 36–60-year-olds. Although the young adult period has a timeframe between late adolescence and 30 s (e.g., Arnett, 2006), there is evidence for Italian young adults of a prolonged delay in reaching developmental tasks related to the adult role, such as entrance into the labor market and the formation of a new family (e.g., Mazzuco et al., 2006; Buhl and Lanz, 2007; De Rose et al., 2008; Mary, 2014; Crocetti et al., 2015). In respect to the adult groups, we considered subjects into a more stable working-age group that was still far from the retirement period. In Table 1 were summarized the sociodemographic characteristics of our sample (e.g., civil status, work position, income).

**TABLE 1:** Sociodemographic characteristics of the sample.

	Total sample		Young adults		Adults	
			
	*n*	%	*n*	%	*n*	%
**Civil status**						
Single	340	25.4	268	36.4	72	11.9
Married	389	29.0	37	5.0	352	58.3
Divorced	42	3.1	-	-	42	6.9
Separated	29	2.1	3	0.4	26	4.3
Cohabiting	225	16.8	156	21.2	69	11.4
In a relationship, but not living together	297	22.1	271	36.8	26	4.3
Widowed	14	1.0	-	-	14	2.3
Other	5	0.4	2	0.3	3	0.5
**Region**						
Northern Italy	200	24.8	104	14.1	96	15.9
Central Italy	809	60.3	467	63.4	342	56.6
Southern Italy	200	14.9	166	22.5	166	27.5
**Education**						
Elementary school	4	0.3	-	-	4	0.7
Middle school	112	8.4	41	5.6	71	11.8
High school	497	37.1	229	31.1	268	44.4
Bachelor degree	250	18.7	203	27.5	47	7.8
Master degree or higher	477	35.6	264	35.8	213	35.4
**Work condition (before the COVID-19 breakdown)**						
Students (i.e., high school or university)	263	19.7	255	33.6	8	1.3
Full-time job	584	43.6	236	32.0	348	57.7
Part-time job	143	10.7	69	9.4	74	12.3
Unemployed	161	12.0	102	13.8	59	9.8
Retirement	8	0.6	-	-	8	1.3
Other (not specified)	181	13.5	75	10.2	106	17.6
**Job loss due to COVID-19 pandemic**						
No	409	79.6	302	79.5	107	79.9
Yes	105	20.4	78	20.5	27	20.1
**Family income**						
Up to 15.000 €	357	27.9	239	34	118	20.6
16.000–50.000 €	717	56.1	369	52.4	348	60.7
51.000–70.000 €	105	8.2	60	8.5	45	7.9
Beyond 71.000 €	98	7.7	36	5.1	62	10.9
**Change in family income related to COVID-19 pandemic**						
It decreased a lot (more than 25%)”	255	19.3	117	16.1	138	23.3
It decreased a little bit (between 5 and 25%)	421	31.9	261	36.0	160	27.0
It did not change at all or it did not significantly change (less than 5%)	603	45.8	330	45.5	273	46.1
It increased a little bit (between 5 and 25%)	35	2.7	18	2.5	17	2.9
It increased a lot (more than 25%)	4	0.3	-	-	4	0.7

### Procedure

Ethical approval by the local Institutional Review Board of the Department of Psychology of Sapienza University of Rome and informed consent from participants were obtained. Data collection was carried out from May until June of the first year of the pandemic, *via* an electronic platform. Links were sent anonymously to participants by trained researchers. Eligibility criteria were the legal age and the formal acceptance of the informed consent. Participation was voluntary and personal information was not included in the dataset. The online survey was filled autonomously by each participant and lasted approximately 25 min.

### Measures

#### Positivity

Positivity was assessed using eight items of the Positivity Scale (Caprara et al., 2012) which measures individuals’ dispositional tendency to view oneself, life, and future under a positive outlook. Items were rated on a 5-point Likert scale, ranging from 1 = “strongly disagree” to 5 = “strongly agree” (i.e., “I look forward to the future with hope and enthusiasm,” or “I am satisfied with my life”). Previous studies attested to the reliability and the validity of the scale (i.e., Caprara et al., 2012; Zuffianò et al., 2019). In the present study, Cronbach’s reliability was α = 0.83, α = 0.86, and α = 0.78 for the total sample, young adults, and adults, respectively.

#### Irritability

Irritability was assessed using four items of the Irritability Scale (Caprara et al., 1985) which measures the personality tendency to react impulsively, aggressively, and rudely at the slightest provocation and disagreement. Items were rated on a 6-point Likert scale, ranging from 1 = “Completely false to me” to 6 “Completely true to me” (i.e., “When I am tired, I easily lose control,” or “I often feel like a powder keg ready to explode”). Previous studies supported the psychometric properties of this instrument (i.e., Caprara et al., 1992; Caprara G. V. et al., 2017). In the present study, Cronbach’s reliability was α = 0.72, α = 0.67, and α = 0.77 for the total sample, young adults, and adults, respectively.

#### Hostile Rumination

Hostile rumination was assessed using five items derived from the Dissipation-Rumination Scale (Caprara, 1986) that measures the extent to which individuals show frequently and prolonged negative antagonistic thoughts after self-threatening provocations, and to experience prolonged negative feelings, expectations, attributions, and desires. Items were rated on a 6-point Likert scale, ranging from 1 = “Completely false to me” to 6 “Completely true to me” (i.e., “I hold a grudge, for a very long time, toward people who have offended me,” or “When I am offended by someone, the more I think about it the angrier I feel”). Previous studies attested the validity and the reliability of the scale across time and countries (i.e., Guzman, 2006; Caprara et al., 2007). In the present study, Cronbach’s reliability was α = 0.77, α = 0.76, and α = 0.80 for the total sample, young adults, and adults, respectively.

#### Regulatory Emotional Self-Efficacy During Quarantine

Self-efficacy beliefs in Expressing Positive Emotions (SE-positive emotion) and in Anger regulation (SE-anger) were assessed using thirteen items adjusted for the purposes of the study from the Emotional Self-Efficacy Beliefs Scale (Caprara and Gerbino, 2001; Bandura et al., 2003) that originally includes two dimensions assessing individual’s perceived capability to express positive emotions and to manage negative emotions. In the present study we asked each participant, during the lockdown period, how well can he/she felt capable to express positive emotions (SE-positive emotions, three items, for example, “Express joy when good things happen to you,” or “Enjoy fully for the good things that happen to you”), or to manage anger (SE-anger, three items, for example, “Get over irritation quickly for wrongs you have experienced,” or “Avoid flying off the handle when you get angry”). Each item was rated on a 5-point Likert scale, ranging from 1 = “not well at all” to 5 = “very well.” Previous studies supported the validity of the instrument across different ages and countries (i.e., Caprara et al., 2008). In the present study, Cronbach’s reliability for SE-positive emotions was α = 0.90, α = 0.91, and α = 0.89 for the total sample, young adults, and adults, respectively, and Cronbach’s reliability for SE-anger were α = 0.77, α = 0.75, and α = 0.79 for the total sample, young adults, and adults, respectively.

#### Prosocial Behavior

Prosocial behaviors during the quarantine were measured using nine items of the Prosocial Behavior Scale (Caprara et al., 2005). In general, this scale was widely used to assess different forms of engaging in prosocial behaviors, such as helping, donating, or sharing things with others. For the purposes of the present study, we asked each participant to focus on the entire lockdown period. Items were assessed on a 5-point Likert scale ranging from 1 = “never/almost never” to 5 = “always/almost always” (i.e., “I try to console those who are sad,” or “I easily put myself in the shoes of those who are in discomfort”). Previous studies supported the psychometric properties of the scale (e.g., Pastorelli et al., 2016). In the present study, Cronbach’s reliability was α = 0.87, α = 0.86, and α = 0.88 for the total sample, young adults, and adults, respectively.

#### Interpersonal Aggression

Interpersonal aggressive behaviors during the quarantine were assessed using the Physical and Verbal Aggression Scale (PVA; Caprara and Pastorelli, 1993; Archer and Coyne, 2005). Overall, the instrument was created to assess a variety of aggressive behaviors, such as hurt, fight, and verbally insulting others, operationalized into the sub-domain of physical, verbal, and indirect aggression. For the purposes of the present study, we considered four items of the verbal aggression sub-scale, asking each participant to focus over the entire lockdown period. Items were assessed on a 5-point Likert scale ranging from 1 = “never/almost never” to 5 = “always” (i.e., “I insult others,” or “I juke others”). Previous research supported the reliability and the validity of this instrument (e.g., Caprara et al., 2001). In the present study, Cronbach’s reliability was α = 0.80, α = 0.82, and α = 0.72 for the total sample, young adults, and adults, respectively.

#### Depressive Symptoms

Depressive symptoms during the quarantine were assessed through the eleven items of the Depression Center for Epidemiologic Studies—Depression Scale (CES-D; Radloff, 1977). Overall, this scale is widely used to measure depressive symptoms of individuals during the last months or the last 2 weeks. For the purposes of the present study, we asked each participant to focus over the last week. Items were assessed on a 4-point Likert scale ranging from 0 = “rarely or never” to 3 = “most of the time” (i.e., “I felt that I could not shake off the blues even with help from my family or friend,” or “I thought my life had been a failure”). A large body of studies supported the psychometric properties of this instrument (e.g., Fava, 1983). In the present study, Cronbach’s reliability was α = 0.90, α = 0.90, and α = 0.89 for the total sample, young adults, and adults, respectively.

#### Anxiety Problems

Anxiety problems during the quarantine were assessed using eight items derived from the State-Trait Anxiety Inventory (STAI; Spielberger et al., 1983). Overall, this instrument is one of the most frequently used measures of anxiety symptoms and problems, both in research and clinical fields. For the purposes of the present study, we considered the state anxiety scale, which assesses how participants feel at the moment in which they collected the survey. Items were assessed on a 4-point Likert scale ranging from 1 = “not at all” to 4 = “completely” (i.e., “I am worried,” or “I feel upset”). A large body of research demonstrated the validity of this measure (e.g., Balsamo et al., 2013). In the present study, Cronbach’s reliability was α = 0.90, α = 0.91, and α = 0.89 for the total sample, young adults, and adults, respectively.

#### Control Variables

##### Gender

Participants were asked to report their gender. Gender was coded 0 for men and 1 for women.

##### Exposure to COVID-19

Participants were asked to report their personal experience with the infection of the COVID-19 virus. Items were in line with the Survey Tool and Guidance COVID-19 published by the World Health Organization (World Health Organization, 2020) and were aimed to assess individuals’ exposure to pandemic risks. Dichotomous items (0 = no 1 = yes) asked much information such as if the participant or some of its relatives (e.g., a member of the family, a friend) was infected by the COVID-19 virus or was dead due to the virus. If a participant answered “no” to all the items was categorized in the group “No exposure to COVID-19” (i.e., 45,8% of the total sample); if a participant answered one or more “yes” was categorized in the group “Exposure to COVID-19” (i.e., 54,2% of the total sample), due to the lowest frequency of “yes” in the overall sample.

### Data Analytic Approach

First, to test our hypothesis, we conducted a series of Latent Profile Analysis models using Mplus 8.4 (Muthén and Muthén, 1998-2017), to identify profiles based on participants’ levels of positivity, irritability, hostile rumination, self-efficacy in expressing positive emotions, and self-efficacy in managing anger in the total sample. This technique was frequently used in order to organize or classify a sample of individuals into several sub-groups mutually exclusive, each with a unique specific distribution and with similar characteristics within groups that are different from the characteristics that define the other groups (Nylund et al., 2007; Lanza and Cooper, 2016). The underlying statistical framework of this approach is the Bayes’ Theorem and the conditional probabilities (Collins and Lanza, 2009; van de Schoot et al., 2014), which provides two types of information for the identification of latent classes or profiles: a nominal variable that represents the categorical membership to belong to a specific latent class/profile; several continuous variables (i.e., one for each latent class/profile) that represent, for each subject, the posterior probabilities to belong to each of the identified latent class/profile (Collins and Lanza, 2009; Lanza and Cooper, 2016). The entire identification process follows estimation mechanisms that aim to maximize the probabilities to classify individuals in the most probable group for them, using the LogLikelihood algorithm with multiple iterations in order to estimate a set of parameters for maximizing the log-likelihood functions (Collins and Lanza, 2009). In order to select the model that best fit the number of profiles in our sample, we compared the 2-, the 3-, and the 4- class models, using the following criteria: (a) The information criterion indices, such as the Akaike Information Criterion (AIC; Akaike, 1973), the Consistent AIC (CAIC; Bozdogan, 1987), the Bayesian Information Criterion (BIC; Schwarz, 1978), the Sample-size Adjusted Bayesian Information Criterion (SABIC; Sclove, 1987), and the Approximate Wight of Evidence Criterion (AWE; Banfield and Raftery, 1993), in which lower values indicate a better model fit; (b) The Bootstrap Likelihood Ratio Test (BLRT; McLachlan, 1987): significant values (*p* < 0.05) indicate that the model with k + 1 classes is better than the k class model; (c) Entropy: a level of 0.06 or higher is considered acceptable (Reinecke, 2006; Asparouhov and Muthèn, 2014); (d) The percentage of each profile: each class had to represent at least 5% of the sample (Speece, 1994); (e) The interpretability of each profile (Wang and Wang, 2012). After the identification of the best latent profiles solution, in order to test if the item-response probabilities were equal across age-groups and to compare the latent profile solution across different age-stages (i.e., the groups showed similar characteristics across age in our sample), we run a multiple-group Latent Profile Analysis in which we compared the best profiles solution into two different age groups: the Young Adulthood group (i.e., 18–35 years old) and Adulthood (i.e., 36–60 years old; Collins and Lanza, 2009). We conditioned profiles’ prevalence and item-response probabilities on the two different age groups, estimating two different sets of prevalence and probabilities of profiles across age, in order to compare the two solutions (Collins and Lanza, 2009). Thus, we examined the invariance of profiles across ages, using a series of multiple-group latent profile analyses (LPAs) with age-groups as the grouping variable, comparing a model in which means of the latent profiles were constrained to be equal across age-groups and a model in which means of the latent profiles were freely estimated in the two different age-groups (Eid et al., 2003). We compared these two different models using the BIC index, in which the lowest values indicate the best model solution (Eid et al., 2003; Collins and Lanza, 2009). Lastly, in order to examine the discriminant contribution of the identified profiles solution, we run a Multivariate Analysis of Variance (MANOVA; Von Eye, 1990), examining the shape of the identified profiles (i.e., the configuration of each profile compared to the other profiles), as well as the level of profiles (i.e., the mean differences among profiles on the indicator variables that we used to identify profiles). We conducted this analysis both on the total sample as well as on each of the two age groups, to analyze characteristics of profiles.

Second, we run a path analysis model within a multiple-group approach, in order to examine associations of latent profiles with concurrent prosocial behaviors, interpersonal aggression, depressive symptoms, and anxiety problems, controlling for participants’ gender and level of exposure to COVID-19. According to previous research (e.g., Broidy et al., 2003; Luengo Kanacri et al., 2014; Favini et al., 2018), as an indicator of latent profiles we considered the posterior probabilities of each individual of being in each latent profile (i.e., the continuous variables). The multiple-group path analysis model was modeled considering age groups as the grouping variable, participants’ gender, and level of exposure to COVID-19 as the two covariates, the latent profiles as predictors, and indicators of prosocial behaviors, interpersonal aggression, depressive symptoms, and anxiety problems as outcomes. We estimated the models using the Robust Maximum Likelihood estimation (MLR; Wang and Wang, 2019), comparing a linear full-constrained model (i.e., a model in which all the estimated parameters were constrained to be equal across groups) with a full-unconstrained model (i.e., a model in which all the parameters were freely estimated across groups) using the Chi-square difference test (Δχ2) with *p* < 0.01; if the difference was significant, we released one parameter at a time, comparing the partially constrained model with the previous model each time, until the Δχ2 was no longer significant (Kline, 1998). In order to evaluate the goodness of fit of the path model, we used the following criteria: χ2 Likelihood Ratio Statistic, the Comparative-Fit Index (CFI) and the Tucker-Lewis-Fit Index (TLI) greater than 0.95 (Hu and Bentler, 1999), the Root Mean Square Error of Approximation (RMSEA) with associated confidence intervals lower than 0.05, and the Standardized Root Mean Square Residual (SRMR) lower than 0.06 (Kline, 2016).

## Results

### Results of the Personality Profiles

**FIGURE 1:**
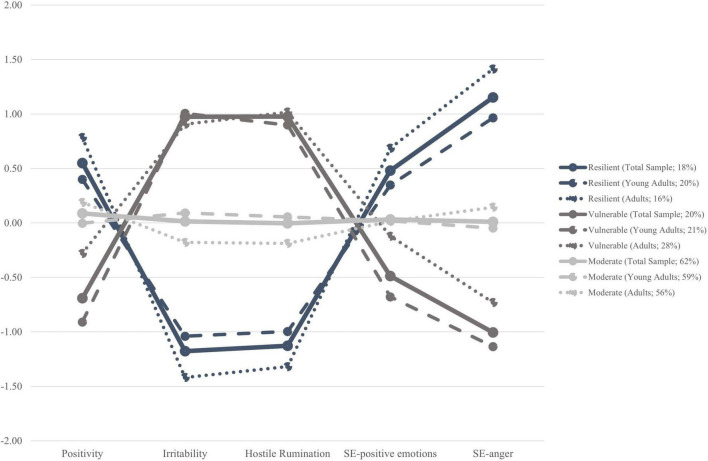
Graphic interpretation of the three emerged personality profiles (i.e., Resilient, Vulnerable, and Moderate) in the total sample, young adults, and adults.

LPA was used to identify personality profiles characterized by broad individual dispositions (i.e., positivity, irritability, and hostile rumination), SE-positive emotions, and SE-anger. The 2-class, 3-class, and 4-class models were compared based on criteria detailed in the Data Analytic Approach section. As shown in Table 2, results indicated that the 3-class model was the model that best fit our data. In detail, the 3-class model identifies three different profile configurated as follow (see Figure 1):

**TABLE 2:** Model fit statistics for the Latent Profile Analysis of the personality profile.

Model	K	-2LL	*npar*	AIC	CAIC	BIC	SABIC	AWE	LRT *p*	Adj LRT *p*	BLRT *p*	Entropy
(1) 2-class	2	−8611.628	16	17255.257	17353.667	17337.668	17286.844	17500.078	<0.001	<0.001	<0.001	0.654
(2) 3-class	3	−8470.890	22	16985.780	17121.095	17099.096	17029.213	17322.411	<0.001	<0.001	<0.001	0.715
(3) 4-class	4	−8387.595	28	16831.190	17003.410	16975.410	16886.468	17259.629	0.671	0.675	<0.001	0.676
(4) 3-class (free)	3	−9305.523	40	18691.045	18937.074	18897.074	18770.014	19303.102				
(5) 3-class (constrained)	3	−9306.406	38	18688.812	18922.539	18884.539	18763.832	19270.265				

*k, number of profiles provided in the model; npar, number of parameters estimated.*

*The following fit indexes are reported: AIC, Akaike Information Criterion; CAIC, Consistent Akaike“s Information Criterion; BIC, Bayesian Information Criterion; SABIC, Sample-Size Adjusted BIC; AWE, Approximate Weight of Evidence Criterion; BLRT, The Bootstrap Likelihood Ratio Test.*

*Significant values (p < 0.05).*

1. The Resilient profile included 18.9% of the sample and was characterized by higher scores of Positivity, SE-positive, and SE-anger and lower scores of Irritability and Hostile Rumination.
2. The Vulnerable profile included 22.0% of the sample and was characterized by higher scores of Irritability and Hostile Rumination and lower scores of Positivity, SE-positive emotions, and SE-anger.
3. The Moderate profile included 59.1% of the sample and was characterized by average scores of all dimensions.

Following the recommendations of Eid et al. (2003), we conducted the measurement invariance of prevalence and item-response probabilities across the two age groups (young adults vs. adults). Results showed that the full constrained model showed a lower BIC (BIC = 18884.539) compared to the freely estimated model (BIC = 18897.074) indicating substantial equality of prevalence and item-response probabilities across the two age-groups, thereby allowing meaningful comparison between young adults and adults profiles in their associations with adaptive and maladaptive outcomes.

Results of the Multivariate Analysis of Variance (MANOVA) allowed us to corroborate the discriminant value of the 3-class solution. In detail, the configuration of each profile significantly differs compared to the other profiles (Wilks’ λ = 0.251, *p* < 0.001), as well as results indicated a significant mean differences among profiles on the indicator variables (i.e., Positivity: [*F*(2,1275) = 147.609, *p* < 0.001], Irritability [*F*(2,1275) = 709.148, *p* < 0.001], Hostile Rumination [*F*(2,1275) = 617.431, *p* < 0.001], SE-positive [*F*(2,1275) = 74.222, *p* < 0.001], and SE-anger [*F*(2,1275) = 673.501, *p* < 0.001]).

### Results of the Multiple-Group Path Analysis Model

To explore whether the identified personality profiles were related with adaptive and maladaptive COVID-related outcomes, we conducted a path analysis with a multiple-group approach, in which the probability of belonging to two (i.e., Resilient and Vulnerable profiles) of the three emerged personality profiles were simultaneously regressed on one indicator of adjustment (i.e., prosocial behavior) and three indicators of maladjustment (i.e., interpersonal aggression, depressive symptoms, and anxiety problems) occurred during the first Italian lockdown. We excluded the Moderate profile because of methodological and theoretical reasons. Methodologically, using the posterior probabilities of group membership means that each individual has a membership probability from 0.00 to 1.00 in each group. Thus, considering all the three posterior probabilities variables would imply a perfect correlation (*r* = 1.00) between the three variables. From a theoretical point of view, considering the average scores on all indicators, one may hypothesize that the Moderate profile would be less informative regarding cross-sectional associations with adaptive and maladaptive COVID-related outcomes, therefore our aim was to analyze more in-depth how specific difficulties in a particular overarching pattern of functioning was associated with specific indicators of adjustment and maladjustment. Moreover, we included two covariates in our path analysis model: gender and exposure to COVID-19.

Overall, across both age groups (i.e., young adults and adults), results showed positive associations between the Resilient profile and prosocial behavior and negative associations with interpersonal aggression, depressive symptoms, and anxiety problems. Contrarily, results indicated negative associations between the Vulnerable profile and prosocial behavior, and positive association with interpersonal aggression, depressive symptoms, and anxiety problems that occurred during the first Italian lockdown.

Results of the multiple-group path analysis model were reported in Table 3. Specifically, we simultaneously constrained all the regression paths to be equal across young adults and adults. However, the significant increase in the Δχ2 [Δχ2 (25) = 53.958, *p* = 0.001] indicated that the tested effects were not equal across the two age groups. Therefore, a closer inspection of the Modification Indexes (MI) suggested releasing some regression path across the two age groups. In detail, we released—one at a time—the effect of the vulnerability profile on anxiety problems (MI = 12.443), interpersonal aggression (MI = 6.016), and depressive symptoms (MI = 7.173). The final partly constrained model was retained since the lack of statistical significance in the Δχ2 compared to the freely estimated model [Δχ2 (22) = 33.899, *p* = 0.050] and the acceptable fit to our data [χ2 (26) = 38.407, *p* = 0.055, CFI = 0.992, TLI = 0.984, RMSEA = 0.028 (90% CI: 0.000, 0.046), SRMR = 0.033]. These released paths indicated that the effect of the vulnerable profile on the maladaptive outcomes (i.e., interpersonal aggression, depressive, and anxiety problems) that occurred during the first lockdown in Italy differed across young adults and adults.

**TABLE 3:** Profile Membership and covariates effects on Prosocial Behavior, Interpersonal Aggression, Depressive Symptoms, and Anxiety Problems during the first Italian Lockdown due to COVID-19 pandemic.

	Prosocial behavior			Interpersonal aggression			Depressive symptoms			Anxiety problems		
				
	b (β)	SE	*p*	b (β)	SE	*p*	b (β)	SE	*p*	b (β)	SE	*p*
**18–35-year-olds**												
(1) Vulnerable profile	−0.180 (−0.093)	0.063	<0.05	0.632 (0.311)	0.085	<0.001	0.590 (0.352)	0.065	<0.001	0.699 (0.387)	0.070	<0.001
(2) Resilient profile	0.234 (0.109)	0.063	<0.001	−0.336 (−0.149)	0.045	<0.001	−0.331 (−0.178)	0.044	<0.001	−0.354 (−0.177)	0.046	<0.001
(3) Gender (0 = men 1 = women)	0.205 (0.139)	0.042	<0.001	−0.131 (−0.085)	0.037	<0.001	0.149 (0.116)	0.030	<0.001	0.161 (0.117)	0.032	<0.001
(4) Exposure to COVID-19 (0 = no 1 = yes)	0.058 (0.043)	0.039	0.136	0.022 (0.015)	0.034	0.512	0.005 (0.004)	0.030	0.876	0.012 (0.009)	0.031	0.697
**36–60-year-olds**												
(1) Vulnerable profile	−0.180 (−0.086)	0.063	<0.05	0.365 (0.216)	0.084	<0.001	0.357 (0.219)	0.076	< 0.001	0.346 (0.205)	0.075	<0.001
(2) Resilient profile	0.234 (0.118)	0.063	<0.001	−0.336 (−0.211)	0.045	<0.001	−0.331 (−0.216)	0.044	<0.001	−0.354 (−0.222)	0.046	<0.001
(3) Gender (0 = men 1 = women)	0.205 (0.146)	0.042	<0.001	−0.131 (−0.116)	0.037	<0.001	0.149 (0.136)	0.030	<0.001	0.161 (0.143)	0.032	<0.001
(4) Exposure to COVID-19 (0 = no 1 = yes)	0.058 (0.042)	0.039	0.136	0.022 (0.020)	0.034	0.512	0.005 (0.004)	0.030	0.876	0.012 (0.011)	0.031	0.697

*Unstandardized (b) and Standardized (β) regression coefficient, standard error (SE), and p-value (p) of b are reported.*

As reported in Table 3, results of the final model showed that gender resulted significantly associated with all adaptive and maladaptive outcomes in both age groups. In detail, women reported significant association with prosocial behavior, depressive symptoms, and anxiety problems, while men resulted associated with interpersonal aggression. Contrary to our expectations, the covariate exposure to COVID-19, which represented a direct or indirect contact with COVID-19 infection, did not show significant associations with either adaptive or maladaptive outcomes during the first Italian lockdown, indicating the prominent role of personality profiles and gender in accounting effects of quarantine on adaptive and maladaptive outcomes.

## Discussion

The present study examined the extent to which personality profiles derived from three broad dispositional tendencies (i.e., positivity, irritability, and hostile rumination) and self-efficacy beliefs in the emotional domain (i.e., self-efficacy in expressing positive emotions and self-efficacy in regulating anger) were associated with adaptive and maladaptive outcomes occurred during the first Italian COVID-19 lockdown (March–June 2020). These associations were also explored with a special focus on differences between young adults (18–35-year-olds) and adults (36–60-year-olds).

Overall, within a person-oriented approach (e.g., Magnusson, 1998; Bergman et al., 2003), we obtained three configurations of personality profiles (i.e., resilient, vulnerable, and moderate) with different dispositional and self-efficacy characteristics that resulted distinctly and uniquely associated with adaptive and maladaptive outcomes during the prolonged isolation of the first Italian lockdown.

Across the three emerged profiles, while the moderate profile was characterized by average-scores in all variables and the most prevalent in our population (i.e., about 60%), both resilient and vulnerable profiles, were found in approximately 20% of the population and were characterized by a specific pattern of functioning.

The resilient profile seems the well-adapted profile as indicated by higher scores of individual strengths such as positivity, self-efficacy in expressing positive emotions, and self-efficacy in regulating anger, and lower scores in irritability and hostile rumination. As a resilient individual is able to endure and recover quickly from difficult circumstances (Newman, 2005), a higher level in the positivity trait and emotional self-efficacy, and a lower dispositional tendency to react impulsively and have prolonged negative feelings under a threatening circumstance, contributes to sustain people in dealing with internal emotional states (e.g., feelings of loneliness, Lakhan et al., 2020) and assume a more agentic role in shaping the course of their life when facing difficulties as the prolonged isolation of the COVID-19 pandemic.

In contrast, the vulnerable profile seems the most compromised profile, because it was characterized by higher scores in irritability and hostile rumination and lower scores in protective factors such as positivity, self-efficacy in expressing positive emotions, and self-efficacy in regulating anger. This configuration was defined as vulnerable because individuals belonging to this profile may be less capable to manage feelings and challenges related to circumstances perceived as threatening as the strong changes determined by the COVID-19 pandemic, due to their higher level of irritability and hostile rumination and a lower control of one’s internal emotional states (i.e., emotional self-efficacy), as well as a lack of a positive cognitive orientation toward life.

Although these personality profiles resulted substantially equal in their prevalence and item-response probabilities across young adults and adults, the vulnerable profile among the young adults showed lower levels of positivity and emotional self-efficacy beliefs, and higher levels of hostile rumination, compared to the same profile among adults, indicating an emotional-related dysregulation among the youngest. This result is not surprising because several studies found greater difficulties in dimensions such as controlling impulsive reactions or being aware of one’s emotions, and ability to respond in accordance with own’s internal emotional states among the youngest populations (e.g., Cole et al., 1994; Gratz and Roemer, 2004; Arnett et al., 2014).

Our findings supported the expected associations of the resilient and vulnerable profiles with adjustment (i.e., prosocial behavior) and maladjustment (i.e., interpersonal aggression, depressive symptoms, and anxiety problems) during the first Italian lockdown, accounting for some differences among young adults and adults. The resilient profile was positively associated with prosocial behavior and negatively associated with interpersonal aggression, depressive symptoms, and anxiety problems, both in young adults and adults. These results are consistent with previous studies attesting the protective role of individual strengths against the challenging circumstance of the COVID-19 pandemic (e.g., Fischer et al., 2021; Reizer et al., 2021). Individuals with a resilient profile possess dispositions and self-efficacy beliefs conducive to a generally positive outlook toward life, to feel capable to express joy and satisfaction and manage anger responses also toward a challenging situation, as well as to tolerate frustrations, to dominate and modulate emotional and behavioral reactions, and a lower tendency to have prolonged negative antagonistic thoughts toward threatening experiences. Coherently with previous studies (e.g., Mojsa-Kaja et al., 2021; Monteiro et al., 2022), the vulnerable profile, which possesses a lack of dispositional strengths and a general tendency to have prolonged negative feelings and thoughts and easily react to provocation, was negatively associated with the enactment of prosocial behavior during quarantine and positively associated with interpersonal aggression, depressive symptoms, and anxiety problems in both age groups.

As regards the differences that emerged between young adults with adults in these associations, we found that young adults were significantly different than adults in the positive association of the vulnerable profile with interpersonal aggression, depressive symptoms, and anxiety problems. Since young adults showed a more compromised vulnerable personality profile than adults belonging to the same profile, the significantly different association between young adults’ probabilities of belonging into the vulnerable profile and higher level of maladaptive outcomes that occurred during the first Italian lockdown attested greater negative consequences of the quarantine for the youngest. As reported elsewhere (e.g., Ahmed et al., 2020; Gao et al., 2020; Huang and Zhao, 2020; Lei et al., 2020; Ozamiz-Etxebarria et al., 2020; Bareeqa et al., 2021), these results indicated a more compromised experience for young adults than adults during the COVID-19 pandemic.

Regarding the effect of gender, no age group difference emerged. For both young adults and adults, gender resulted statistically significant in predicting prosocial behavior, interpersonal aggression, depressive symptoms, and anxiety problems. Thus, in line with previous studies on differences across gender of the COVID-19 psychosocial effect (e.g., Lei et al., 2020; Mazza et al., 2020; Parola et al., 2020), our results showed that young adults and adults’ women showed a greater tendency to engage in prosocial behaviors, and to experience depression and anxiety symptoms, while young adults and adults’ men showed a greater tendency to engage in interpersonal aggression.

Finally, although we did not find any significant effect of the exposure to COVID-19 in our sample, it is important to consider this result with caution. In the present study, we assessed the exposure to COVID-19 by using a checklist about direct or indirect contact with the COVID-19 infection during the first Italian lockdown. Even if 54,2% of the total sample answered “yes” in one or more statements, this reflected both direct or indirect exposure to the virus. Thus, it is possible to assume a heterogeneity across direct or indirect exposure that did not allow us to capture experience with the virus with a greater level of stress (e.g., impairment symptoms conditions due to the contagious or a relative’s death).

Overall, the present study contributes to further understanding how different configurations of personality profiles resulted associated with adaptive and maladaptive outcomes that occurred during the prolonged isolation lived in Italy from March to June 2020. To our knowledge, this is the first study during the COVID-19 pandemic that used a person-oriented approach to identify groups with different levels of individual characteristics that may mitigate or exacerbate the psychological effect of the COVID-19 lockdown in Italy. Moreover, the present study offered further evidence regarding the greater maladaptive consequences of COVID-19 for vulnerable young adults. Thus, in times of pandemia, intervention actions that promote the capacity to regulate negative emotions in vulnerable young adults should be a priority. Moreover, our results have also important implications for future studies not related to the pandemic era. Previous studies that explored individual functioning under the person-oriented approach mostly focused on Big Five personality profiles (e.g., Favini et al., 2018) or multi-faceted aspects of individual characteristics (e.g., identity exploration, Crocetti et al., 2015). The present study is the first to consider more malleable characteristics as individual dispositions and individual perceptions of agency and capabilities (i.e., self-efficacy beliefs) under a person-oriented approach.

### Limitations and Future Directions

Despite different strengths, the present study has some limitations that should be taken into consideration. First, although our large sample included 1341 participants, approximately 70% were women and a small percentage (i.e., 15%) was in the north of Italy, which was the Italian area most affected by the COVID-19 contagious spread during the first wave. Moreover, our results might be biased due to the use of a non-probabilistic sampling method (e.g., convenience sample, snowball sample). Thus, the generalizability of our results could be affected by these unbalanced sociodemographic characteristics and the sampling method. Second, the cross-sectional design of our study did not allow us to explore the predictive role of personality profiles on the development of adaptive and maladaptive outcomes. Moreover, considering the partly malleable nature of individual dispositions (e.g., Roberts et al., 2008) and the domain and time specificity of self-efficacy beliefs (Bandura, 1997), we could not control for any possible changes across levels of individual dispositions and self-efficacy beliefs due to the prolonged isolation period. Future studies should cover these gaps by using a longitudinal design that could clarify the predictive role of personality profiles on adjustment and maladjustment during the pandemic, as well as to capture possible changes of personality profiles related to the COVID-19 outbreak. Finally, the present study used self-report measures that might be affected by social desirability.

## Conclusion

The present study demonstrates the importance to consider a holistic perspective of individual functioning in the examination of psychological consequences of the COVID-19 pandemic. Furthermore, we evidenced that in a condition of high stress, such as the COVID-19 emergency in Italy, the Resilient individuals are better protected and adjusted, while vulnerable young people are at risk of psychological and psychosocial maladjustment. The transition to adulthood is a period of major biological, psychological, and social changes, characterized by opportunities and challenges that can have long-term implications. The emergence of the pandemic might have further jeopardized this life transition. We have contributed to the identification of the vulnerable young adult group, and this is an important step for the development of preventive and promotion actions. Vulnerable young adults with less personal resources are more likely to experiment uncertainty and worry about decisions related to their formative period and work, have less hope in the future, and are less able to regulate their thought and manage their emotions. We do not know yet when the COVID-19 pandemic will be over, but we think that it is crucial for researchers and health professionals to prioritize individuals belonging to the vulnerable group.

## Data Availability Statement

The raw data supporting the conclusions of this article will be made available by the corresponding author on request, without undue reservation.

## Ethics Statement

The studies involving human participants were reviewed and approved by Local Institutional Review Board–Department of Psychology, Sapienza University of Rome (Rome, Italy). The patients/participants provided their written informed consent to participate in this study.

## Author Contributions

FC conceived of the study, participated in its design, drafted the manuscript, performed the statistical analysis, and interpreted the data. CP conceived of the study, participated in its design, drafted the manuscript, and interpreted the data. AF participated in the study design, drafted the manuscript, and performed the statistical analysis. CR conceived of the study, participated in its design, and drafted the manuscript. AZ contributed on interpretation of the data, and drafted and revised the manuscript. EB participated in the study design, contributed on interpretation of the data, and drafted and revised the manuscript. ET conceived of the study, participated in its design, contributed on interpretation of the data, and revised the manuscript. MG contributed on interpretation of the data and revised the manuscript. FG contributed to drafting and revising the manuscript. All authors read and approved the final manuscript.

## Conflict of Interest

The authors declare that the research was conducted in the absence of any commercial or financial relationships that could be construed as a potential conflict of interest. The reviewer RB declared a shared affiliation with the authors to the handling editor at the time of the review.

## Publisher’s Note

All claims expressed in this article are solely those of the authors and do not necessarily represent those of their affiliated organizations, or those of the publisher, the editors and the reviewers. Any product that may be evaluated in this article, or claim that may be made by its manufacturer, is not guaranteed or endorsed by the publisher.
